# Supplementary material for: Differential Amperometric Microneedle Biosensor for Wearable Levodopa Monitoring of Parkinson’s Disease
Source: Biosensors (Basel). 2022 Feb 7;12(2):102. doi: 10.3390/bios12020102 (PMC8869619; doi:10.3390/bios12020102)
Supplement: Supplementary file 1 [file biosensors-12-00102-s001.zip › biosensors-1583046-supplementary.pdf]

biosensors MDPI

Article

Differential Amperometric Microneedle Biosensor for Wearable Levodopa Monitoring of Parkinson's Disease

Lu Fang [1,†], Hangxu Ren [2,†], Xiyu Mao [2], Shanshan Zhang [2], Yu Cai [2], Shiyi Xu [2],Yi Zhang [1], Lihua Li [1,*], Xuesong Ye [2,*], Bo Liang [2,*]

[1] Department of Automation, Hangzhou Dianzi University , Hangzhou 310018, China; Fanglu@hdu.edu.cn (L.F.); 20061135@hdu.edu.cn (Y.Z.)
[2] Biosensor National Special Laboratory, Key Laboratory of Biomedical Engineering of Ministry of Education, College of Biomedical Engineering & Instrument Science,Zhejiang University, Hangzhou 310058, China; 21815097@zju.edu.cn (H.R.); 21915002@zju.edu.cn (X.M.); 12015019@zju.edu.cn (S.Z.); 3130104519@zju.edu.cn (Y.C.); xushiyi@zju.edu.cn (S.X.)
* Correspondence: lilh@hdu.edu.cn (L.L.); yexuesong@zju.edu.cn (X.Y.); boliang1986@zju.edu.cn (B.L.); Tel.: +86-571-86878587 (L.L.); Tel.: +86-571-87952756 (X.Y. & B.L.)
† These authors contributed equally to this work.

1. Material and Methods

1.1. Materials and Reagents

PTyrosinase (TYR, 1190 U/mg) were purchased from Solarbio (Beijing, China). Fetal bovine serum (FBS) was provided by CellMax (Beijing, China). PBS solution (0.01 M, pH = 7.4) was purchased from Sangon Biotech (Shanghai, China). Bovine serum albumin (BSA), ascorbic acid (AA), uric acid (UA), glucose, and chloroauric acid were purchased from Aladdin Co., Ltd. (Shanghai, China). Polyurethane (PU) was purchased from Sigma-Aldrich. Aluminum oxide polishing powder (d=0.05 μm) was purchased from HWRK Chem (Beijing, China), Other reagents were purchased from Sinopharm Chemical Reagent Co., Ltd. (Beijing, China) and were used without further modification. Male Sprague-Dawley rats were provided by Ziyuan Experimental Animal Technology Co., Ltd (Hangzhou, China).

1.2. Instruments and in Vitro Evaluation

PScanning electron microscopic (SEM) images were taken on a field emission scanning electron microscope (ZEISS, GeminiSEM 300, Germany) at an accelerating voltage of 3.0 kV, combined with Energy Dispersive X-Ray Spectroscopy (EDS) for elemental compositions analysis.

The electrochemical performance of the modified working electrode WE1 and WE2 were evaluated in PBS and bovine serum. CV was used to test the electrochemical behaviors of the working electrode in 0.01 M PBS solution with the potential window of -1.0 V to 0.8 V at the scan rate of 100 mV/s. Amperometric response of the electrode to different concentrations of L-dopa was measured in 0.01 M PBS and bovine serum under the applied potential of 0.3 V. Anti-interference test was carried out in PBS by adding interfering substances at the physiologically relevant concentrations. The long-term stability of the two electrodes was also tested. WE1 and WE2 were stored in 0.01 M PBS at 4 ^{o}C for two weeks and the amperometric response of the electrode was tested every few days during storing. All these electrochemical measurements were done on an electrochemical workstation (Multi Autolab M204, Metrohm, Switzerland) at room temperature, with an Ag/AgCl reference electrode (3 M KCl, Chenhua Electronic Technology, China) and a platinum counter electrode (0.5 mm in diameter, Chenhua Electronic Technology, China).

The performance of FDMA was evaluated by chronoamperometry in bovine serum containing L-Dopa. A layer of rat skin covered on the surface of serum to simulate the in

vivo environment and the percutaneous detection capability of the electrodes was tested. The collected rat skin was stored at -20 oC before use, then, it was thawed at room temperature and cut into a suitable size with a scalpel. During tests, the FDMA was carefully pierced through the skin, exposing a tip of 3mm soaked in the serum for testing. The chronoamperometry response of the FDMA was tested with the dual channel electrochemical workstation (Multi Autolab M204, Metrohm, Switzerland) at room temperature at the applied potential of 0.3 V.

1.3. In Vivo Evaluation

In order to evaluate the in-vivo performance of the electrode, the FDMA was implanted subcutaneously of a male Sprague-Dawley rat (200 g, provide by Ziyuan Laboratory Animal Technology Co., Ltd., Hangzhou, China) for continues monitoring. The implantation process was as follows: first, the assembled sensor was sterilized with gamma irradiation (16 Gy) before implantation. Then, the rat is anesthetized by injecting 3% pentobarbital sodium into the abdominal cavity. Next, the hair on the back of the neck was shaved and the exposed skin was surgically disinfected with 75% ethyl alcohol and iodine tincture. After that, the assembled sensor was implanted under the skin and medical tape was used to fix the sensor and prevent it from falling during moving.

The dual channel electrochemical workstation (Multi Autolab M204, Metrohm, Switzerland) was used to measure the in-vivo response of the sensor. One channel was connected to electrode group 1 (WE1, CE1 and RE1), and the other channel was connected to electrode group 2 (WE2, CE2 and RE2). During measurement, a constant potential of 0.3 V was applied on both group and the response signal of the sensor was continuously recorded. After about 2500 seconds, the signal was stable, then 4 ml of 8 mM L-Dopa solution was injected into the rat's abdominal cavity to evaluate the in vivo response of the sensor. After that, we continuously recorded the sensor signal from rising to recovering for about 2500 seconds. After the experiment, the rat was sacrificed under anesthesia.

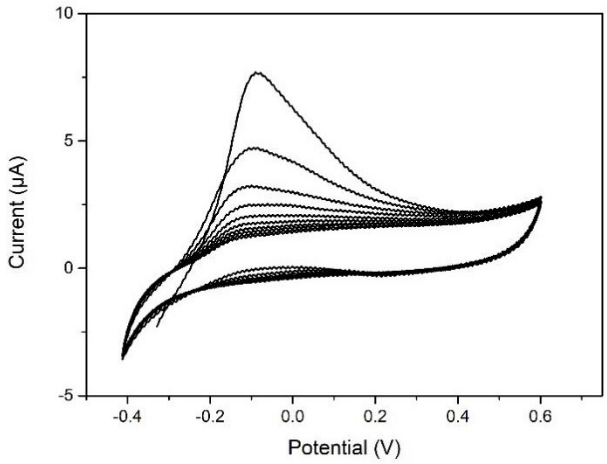

Figure S1. Cyclic voltammetry for stainless steel microneedle during the electrochemical cleaning process: cyclic voltammetry scan range: -0.4 V to 0.6 V, 10 cycles, scan rate 50 mV/s.

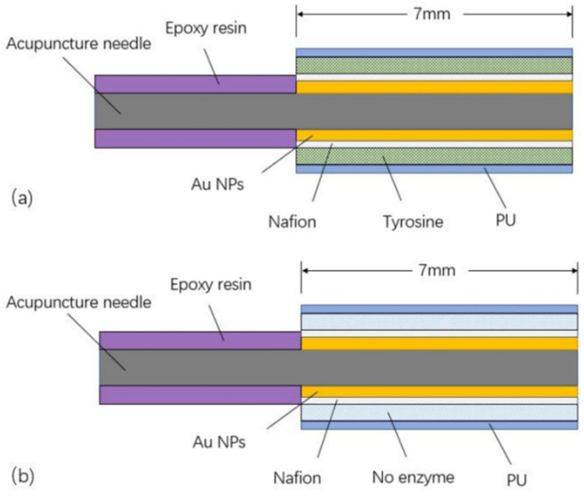

Figure S2. Structure of the working electrode WE1 and WE2.

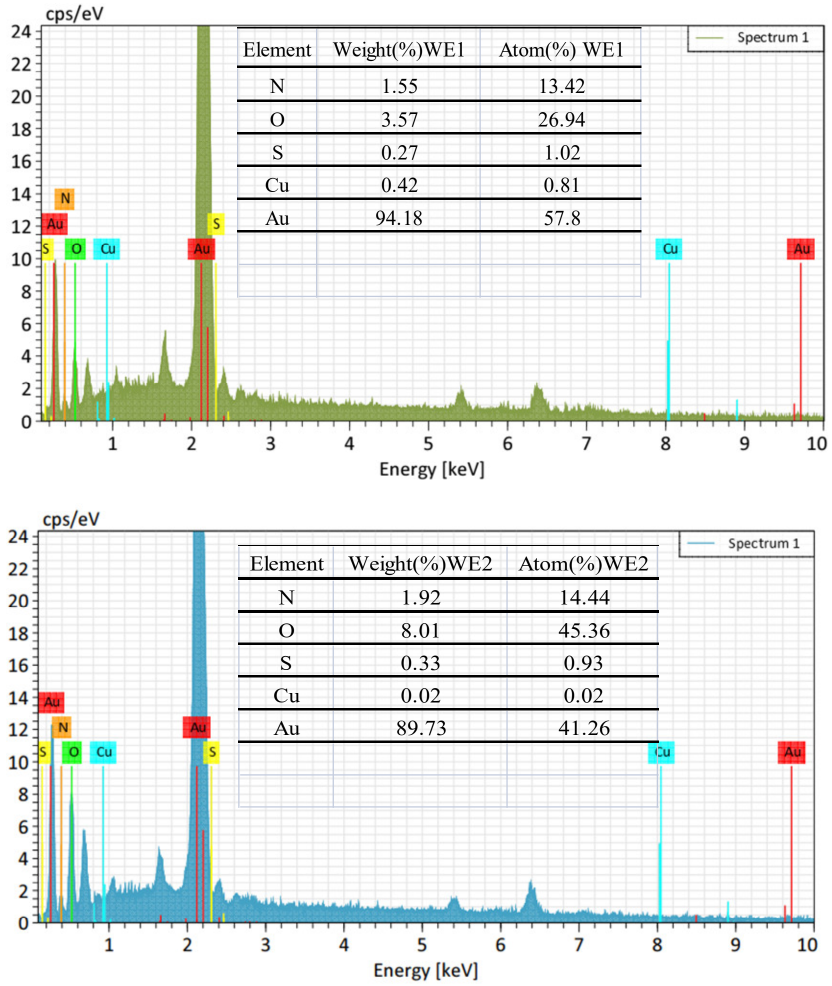

Element	Weight(%)WE1	Atom(%) WE1
N	1.55	13.42
O	3.57	26.94
S	0.27	1.02
Cu	0.42	0.81
Au	94.18	57.8

Element	Weight(%)WE2	Atom(%)WE2
N	1.92	14.44
O	8.01	45.36
S	0.33	0.93
Cu	0.02	0.02
Au	89.73	41.26

Figure S3. EDS elemental composition analysis of the two working electrodes WE1 and WE2.

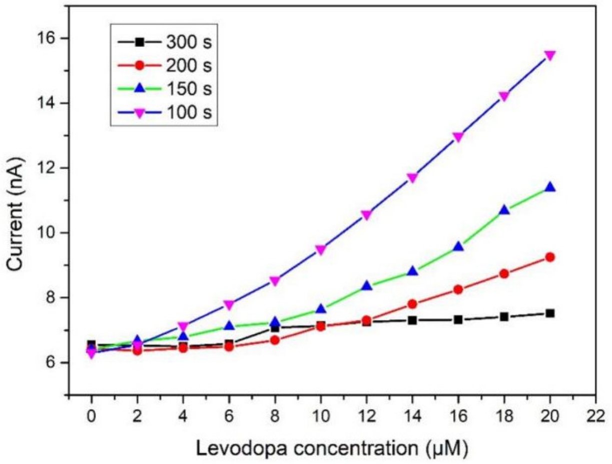

Figure S4. Current response curve of WE2 to L-Dopa with different PANI electropolymerization time.

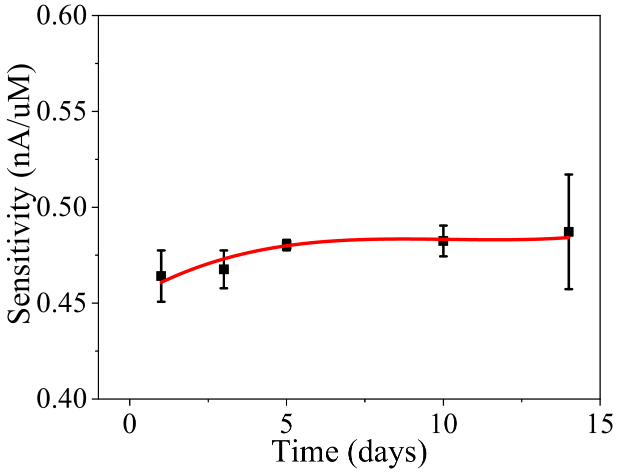

Figure S5. The sensitivity fluctuation of WE2 to L-Dopa during two weeks storage in 0.01 M PBS at 4 °C.
